# Anti-Inflammatory and Neuroprotective Effects of Constituents Isolated from *Rhodiola rosea*

**DOI:** 10.1155/2013/514049

**Published:** 2013-04-16

**Authors:** Yeonju Lee, Jae-Chul Jung, Soyong Jang, Jieun Kim, Zulfiqar Ali, Ikhlas A. Khan, Seikwan Oh

**Affiliations:** ^1^Department of Neuroscience and Tissue Injury Defense Research Center, School of Medicine, Ewha Womans University, Seoul 158-710, Republic of Korea; ^2^National Center for Natural Products Research, School of Pharmacy, Thad Cochran Research Center, University of Mississippi, MS 38677-1848, USA

## Abstract

To determine the biological activity of *Rhodiola rosea*, the protein expression of iNOS and proinflammatory cytokines was measured after the activation of murine microglial BV2 cells by LPS under the exposure of constituents of *Rhodiola rosea*: crude extract, rosin, rosarin, and salidroside (each 1–50 **μ**g/mL). The LPS-induced expression of iNOS and cytokines in BV2 cells was suppressed by the constituents of *Rhodiola rosea* in a concentration-dependent manner. Also the expression of the proinflammatory factors iNOS, IL-1**β**, and TNF-**α** in the kidney and prefrontal cortex of brain in mice was suppressed by the oral administration of *Rhodiola rosea* crude extract (500 mg/kg). To determine the neuroprotective effect of constituents of *Rhodiola rosea*, neuronal cells were activated by L-glutamate, and neurotoxicity was analyzed. The L-glutamate-induced neurotoxicity was suppressed by the treatment with rosin but not by rosarin. The level of phosphorylated MAPK, pJNK, and pp38 was increased by L-glutamate treatment but decreased by the treatment with rosin and salidroside. These results indicate that *Rhodiola rosea* may have therapeutic potential for the treatment of inflammation and neurodegenerative disease.

## 1. Introduction

*Rhodiola rosea* (*R*. *rosea*) is known as a golden or arctic root and belongs to the plant family of Crassulaceae, subfamily of Sedoideae, and genus Rhodiola [1]. *R*. *rosea* is widely distributed in the Arctic and mountainous regions throughout Europe and Asia. It is a popular plant in traditional medical systems and has been used to stimulate the nervous system, decrease depression, enhance work performance, and prevent high altitude sickness [2]. Of the Rhodiola species, *R*. *rosea* has been extensively studied for its phytochemical and toxicological properties [3]. *R*. *rosea* root contains about 28 compounds, of which salidroside (rhodioloside), rosavins, and p-tyrosol are thought to have the most critical therapeutic activity [4]. It was reported that *R*. *rosea* ingestion can improve cognitive function [5], reduce mental fatigue [6, 7], promote free radical mitigation, have antioxidative [8] and neuroprotective [9] effects, increase endurance performance [10, 11], and enhance learning and memory [11]. *R*. *rosea* may play a role in the amelioration of neurodegenerative diseases, such as Alzheimer's disease (AD), via its anti-inflammatory and neuroprotective properties.

Alzheimer's disease is the common neurodegenerative disease characterized by the inflammation and neuronal loss in the specific regions of the forebrain. Therefore, any compound that has the antineurotoxicity and the anti-inflammatory properties can be a good candidate for AD therapy. The central nervous system includes two major cell types, neurons, and glial cells; glial cells are represented by astrocytes, oligodendrocytes, and microglia [12]. Once the microglia is activated by lipopolysaccharide, the affected microglia can produce a series of proinflammatory and cytotoxic factors, such as tumor necrosis factor- (TNF-) *α* and interleukin- (IL-) 1*β*, which have been implicated in the neuropathogenesis of AD [13]. Moreover, the release of these proinflammatory factors from the activated cells enhances LPS-induced cytotoxic activations [14] and develops a continuous cycle of inflammatory stimuli. 

L-Glutamate (L-glu) is the most abundant excitatory neurotransmitter in the vertebrate central nervous system (CNS) and plays a crucial role in the neurological processes including cognition, learning, and memory [15]. However, excessive stimulation of glutamate receptors, under pathophysiological conditions, leads to the neuronal damage and death. This phenomenon is well known as “excitotoxicity,” since neurotoxicity is correlated with excitatory properties of various L-glu analogs [16]. Glutamate neurotoxicity has also been postulated to play important roles in the pathophysiology of numerous neurological diseases including hypoxic-ischemic brain injury [17, 18], epileptic seizures [19], and neurodegenerative diseases including AD [20, 21] and Parkinson's disease [22, 23]. Accordingly, substances which can prevent L-glu-induced neurotoxicity are expected to be potential tools in the therapy of various neurological and neurodegenerative diseases. If one compound reduces the L-glu-induced neurotoxicity, it would become a candidate means of improving the neurodegenerative diseases. This experiment was aimed to investigate the effects of the various constituents of *R*. *rosea* on inflammation and neurotoxicity.

## 2. Materials and Methods

### 2.1. Reagents

**Figure 1:**
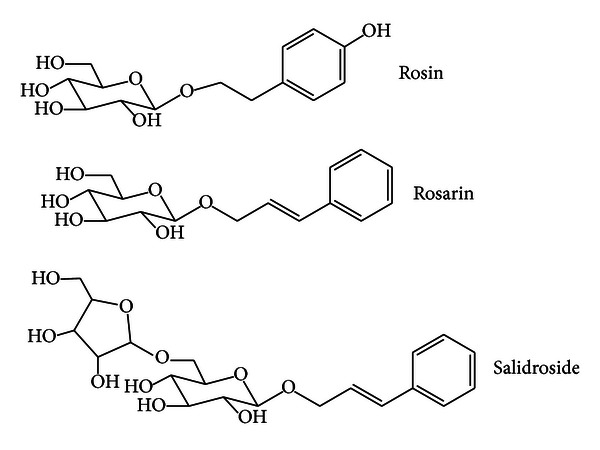
The structures of a constituent of *R*. *rosea*.

LPS (*Escherichia coli*, O111:B4) were obtained from Sigma-Aldrich (St. Louis, MO, USA). Cell culture ingredients were obtained from Invitrogen (Carlsbad, CA, USA). All other reagents were obtained from Sigma-Aldrich. Roots' water extract (230 g) of *Rhodiola rosea* was soaked in MeOH at room temperature. The soluble part was evaporated under reduced pressure to afford a dry brown material (extract, 165 g) and subjected to vacuum liquid chromatography over flash silica gel. Through column fraction, rosin (322 mg), rosarin (339 mg), and salidroside (908 mg) were purified and identified as described in detail [24]. *R*. *rosea* extract and constituents (Figure 1) were kindly supplied from Dr. Ikhlas Khan (NCNPR, University of Mississippi, MS, USA). 

### 2.2. Cell Culture

The murine BV2 cell line (a generous gift from W. Kim, Korea Research Institute of Bioscience and Biotechnology, Daejeon, Republic of Korea), which is immortalized after infection with a *v*-*raf/v*-*myc *recombinant retrovirus, exhibits the phenotypic and functional properties of reactive microglial cells. BV2 cells were maintained at 37°C at 5% CO_2_ in Dulbecco's modified Eagle's medium (DMEM) supplemented with 10% FBS, 100 *μ*g/mL streptomycin, and 100 U/mL penicillin. BV2 cells were grown in 24-well plates at a concentration of 1 × 10^5^ cells/well followed by proper treatment.

### 2.3. Nitrite Assay

NO production from activated microglial cells was determined by measuring the amount of nitrite, a relatively stable oxidation product of NO, as described previously [25]. Cells were incubated with or without LPS in the presence or absence of various concentrations of compounds for 18 h. The nitrite accumulation in the supernatant was assessed by the Griess reaction. In brief, an aliquot of the conditioned medium 50 *μ*L was mixed with an equal volume of 1% sulfanilamide in water and 0.1% *N*-1-naphthylethylenediamine dihydrochloride in 5% phosphoric acid. The absorbance was determined at 540 nm in an automated microplate reader.

### 2.4. Immunoblot Analysis

BV2 and cortical neuronal cells were washed twice with ice-cold phosphate-buffered saline (PBS) and then lysed in ice-cold modified lysis buffer (150 mM NaCl, 50 mM Tris, 1 mM EDTA, 0.01% Triton X-100, and protease inhibitors, pH 8.0), and cellular debris was cleared by centrifugation. Samples were assayed for protein concentration using bicinchoninic acid reagents (Pierce Chemical, Rockford, IL, USA). The supernatants were aliquoted and stored at −70°C until use. Proteins were separated by SDS-polyacrylamide gel electrophoresis and transferred to a polyvinylidene difluoride membrane. The membrane was blocked with 5% skim milk in Tris-buffered saline/Tween 20 solution. The blots were incubated with the TNF-*α*, IL-6, and iNOS (Cell Signaling Technology Inc., Danvers, MA, USA). GAPDH (Santa Cruz Biotechnology, Inc., Santa Cruz, CA, USA) was performed as an internal control. After washing with Tris-buffered saline/Tween 20, horseradish peroxidase-conjugated secondary antibodies (Cell Signaling Technology Inc. Danvers, MA, USA) were applied, and the blots were developed using the enhanced chemiluminescence detection kit (GE Healthcare, Chalfont St. Giles, Buckinghamshire, UK).

### 2.5. Animals

All the experiments were carried out using male ICR mice weighing 28–30 g purchased from the Orient Co., Ltd. (Seoul, Republic of Korea), according to the guidelines of the Animal Care and Use Committee of the School of Medicine, Ewha Womans University in Seoul, Republic of Korea. The mice were housed 6 or 8 per cage, allowed access to water and food *ad libitum*, and maintained at an ambient temperature of 23°C with 40–50% humidity and a 12 h diurnal light cycle (light on 07:00–19:00). Seven-week-old ICR male mice were injected with saline or LPS from *Escherichia coli* (0111:B4, Sigma-Aldrich, St. Louis, MO, 1 mg/kg). LPS was dissolved in saline and injected intraperitoneally. *R*. *rosea* crude extract (500 mg/kg) was administered orally 1 h before LPS injection. Control animals were injected with equivalent volumes of saline. The tissue was collected from mice after 6 h of kidney and 16 h for frontal cortex of brain LPS injection, and changes of proinflammatory cytokine expression were measured by PCR.

### 2.6. Mixed Cortical Culture

After CO_2_ anesthesia, cerebral cortices were removed from the brains of 16-day-old ICR fetal mice. The neocortices were triturated and plated on 24-well plates (with approximately 1 × 10^6^ cells/well), which were precoated with 100 *μ*g/mL poly-D-lysine and 4 *μ*g/mL laminin in modified Eagle's medium (MEM) and supplemented with 5% horse serum, 5% fetal bovine serum (FBS), 2 mM glutamine, and 20 mM glucose. After 6 days in vitro (DIV), the cultures were shifted to the plating media containing 10 *μ*M cytosine arabinoside without FBS. The cultures were then fed twice per week. After 12 to 13 days, more than 90% of neurons were MAP2-positive to immunocytochemical staining and sat on the top of a confluent monolayer of astrocytes. Mixed cortical cell cultures containing neurons and glia (DIV 12–14) were exposed to the excitatory amino acid, L-glutamate, in MEM without 10% horse serum for 24 h to measure the condition of the cells.

### 2.7. Measurement of Neurotoxicity

Cell death was assessed by measuring the activity of lactate dehydrogenase (LDH) released in the culture medium according to the method described by Koh and Choi [26]. Culture medium collected after 18 to 24 h drug treatment was used unless otherwise indicated. An aliquot of 25 *μ*L of culture medium was transferred to a microplate, and 100 *μ*L of NADH solution (0.3 mg/mL NADH and 0.1 M potassium phosphate, pH 7.4) was added to the medium. After 2 min, 25 *μ*L of pyruvate solution (22.7 mM pyruvate and 0.1 M potassium phosphate, pH 7.4) was added. After adding the pyruvate solution, the decrease in absorbance at 340 nm, indicating the conversion of NADH to NAD, was measured using a SpectraMax microplate reader (Molecular Devices, Sunnyvale, CA, USA). LDH activity was normalized on the basis of the reference scale such that the sham-treated culture and culture showing complete cell death were taken as 0 and 100%, respectively, and normalized LDH activity was regarded as an indicator of cell death. 

### 2.8. Polymerase Chain Reaction

ICR mice were administered with LPS in the absence or presence of crude extract of *R*. *rosea* for 6 h or 16 h. Total RNA was isolated from kidney or prefrontal cortex of brain of ICR mice using TRIzol (Invitrogen, CA, USA) according to the manufacturer's instructions. For cDNA synthesis, 2 *μ*g of total RNA was reverse-transcribed using the SuperScript First-Strand Synthesis System (Invitrogen, CA, USA). cDNA was amplified by polymerase chain reaction (PCR) using primers for iNOS (F: GTGTTCCACCAGGAGATGTTG, R: CTCCTGCCCACTGAGTTCGTC), IL-1*β* (F: AGCAACGACAAAATACCTGT, R: CAGTCCAGCCCATACTTTAG), and IL-6 (F: CCACTTCACAAGTCGGAGGC, R: CCAGCTTATCTGTTAGGAGA). Thermal cycling conditions included initial denaturation at 95°C for 2 min, followed by 30 cycles of 1 min at 95°C, 1 min at 56°C and 30 s at 72°C, and then a dissociation step. PCR products were separated by 1% agarose gel electrophoresis and visualized by ethidium bromide staining.

### 2.9. Statistical Analysis

All values were expressed as mean ± S.E.M., and comparisons between groups were performed using analysis of variance followed by the Student-Newman-Keuls test for multiple comparisons. The results are representative of three independent experiments done. Differences with *P* < 0.05 and *P* < 0.01 were considered as statistically significant.

## 3. Results

### 3.1. *Rhodiola rosea* Constituents Suppressed the LPS-Induced NO Generation and iNOS Expression in Microglia

**Figure 2:**
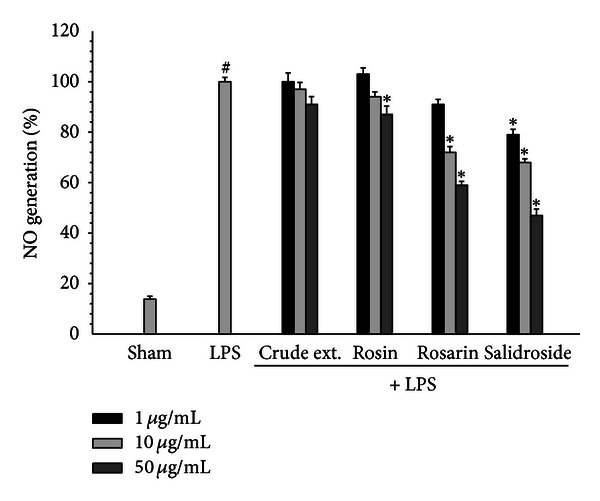
The suppression of NO generation in LPS-treated BV2 microglial cells. Cells were treated with 100 ng/mL LPS with or without *R*. *rosea* constituents (1, 10, 50 *μ*g/mL) for 18 h. At the end of incubation, 50 *μ*L of the medium was collected to measure nitrite production. The amount of NO in the supernatant fractions was measured by using the Griess reagent. All values are expressed as mean ± S.E.M. from three independent experiments. Data were analyzed by one-way ANOVA for multiple comparison and Student-Newman-Keuls test as post hoc test. ^#^ *P* < 0.01 as compared with the vehicle group; *P < 0.05 as compared with the LPS-treated group.

**Figure 3:**
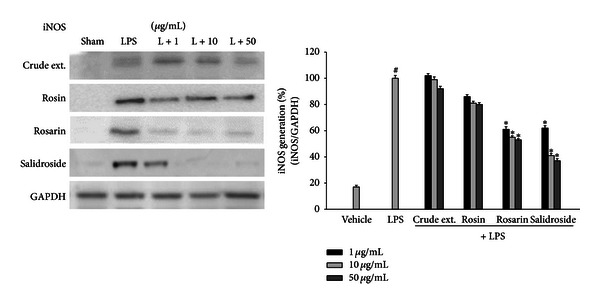
Effects of *R*. *rosea* constituents on iNOS protein expression in LPS-treated microglial cell. BV2 microglial cells were treated with the *Rhodiola rosea* constituents (1, 10, 50 *μ*g/mL) 30 min prior to activation by 100 ng/mL LPS. The protein was collected after 18 h. The iNOS protein levels were measured using immunoblot analysis. *R*. *rosea* active components suppressed the LPS-induced expression of iNOS protein in activated microglia. Results are representative of three independent experiments. Data were analyzed by one-way ANOVA for multiple comparison and Student-Newman-Keuls test as post hoc test. ^#^ *P* < 0.01 as compared with the vehicle group; *P < 0.05 as compared with the LPS treated group.

To investigate the anti-inflammatory effect of *R*. *rosea* constituents, the LPS-induced production of NO was measured in the presence or absence of constituents of *R*. *rosea* in BV2 microglial cells. Microglial cells were treated with *R*. *rosea* constituents 30 min prior to the LPS treatment for 18 h. The constituents, rosarin and salidroside, suppressed the generation of NO in activated microglia in a dose-dependent manner (Figure 2). These findings suggest that *R*. *rosea* constituents may suppress the LPS-induced inflammatory response through the inhibition of NO generation. The expression of iNOS protein was highly induced by LPS, and this expression was inhibited by rosarin and salidroside as did in NO generation (Figure 3). These results implied that suppression of NO generation by *R*. *rosea* might be due to the inhibition of iNOS protein expression by components of *R*. *rosea*.

### 3.2. *Rhodiola rosea* Constituents Reduced the LPS-Induced Expression of Proinflammatory Cytokines

**Figure 4:**
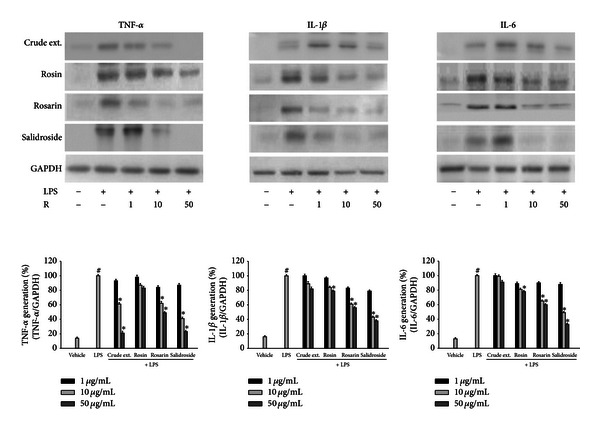
Effects of *R*. *rosea* constituents on the LPS-induced expressions of TNF-*α*, IL-1*β*, and IL-6 in microglial cells. BV2 microglial cells were treated with the *Rhodiola rosea* constituents (1, 10, 50 *μ*g/mL) 30 min prior to activation by 100 ng/mL LPS for 18 h. Cell extracts were collected from cultured microglia after activation by LPS with treatment of *R*. *rosea* constituents, and immunoblot analysis was performed using TNF-*α*, IL-1*β*, and IL-6. *R*. *rosea* suppressed the expression of TNF-*α*, IL-1*β*, and IL-6 in activated microglia, respectively. GAPDH was used as an internal control. Results are representative of three independent experiments. Data were analyzed by one-way ANOVA for multiple comparison and Student-Newman-Keuls test as post hoc test. ^#^ *P* < 0.01 as compared with the vehicle group; *P < 0.05 as compared with the LPS treated group.

Constituents of *R*. *rosea* exerted an anti-inflammatory effect on LPS-induced responses accompanied by the expression of proinflammatory cytokines. Microglial cells were treated with constituents of *R*. *rosea* and LPS for 18 h. The expression levels of the proinflammatory cytokines, TNF-*α*, IL-1*β*, and IL-6 were reduced by the treatment with constituents of *R*. *rosea* in dose-dependent manners (Figure 4). The crude extract of *R*. *rosea* showed suppressing activity to the LPS-induced TNF-*α* expression but did not inhibit the IL-1*β* or IL-6 expression. These results indicated that constituents of *R*. *rosea* (rosin, rosarin, and salidroside) showed the suppression of LPS-induced expression of proinflammatory cytokines in microglia.

### 3.3. *Rhodiola rosea* Extract Reduced the LPS-Induced Elevation of Proinflammatory Cytokine Expression in Mice

**Figure 5:**
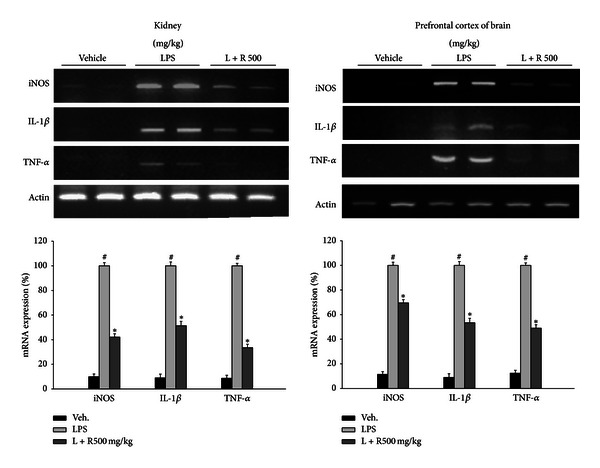
Effects of *R*. *rosea* extract on cytokines mRNA expression in LPS-treated mice. ICR mice were orally administered with the *R*. *rosea* extract (500 mg/kg); 30 min later mice were injected (i.p.) by 1 mg/kg LPS. Expressions of iNOS, IL-1*β*, and TNF-*α* were measured by PCR analysis at 6 h or 16 h after LPS treatment. *R*. *rosea* extract suppressed the expression of iNOS, IL-1*β*, and TNF-*α* in mice 6 h later for kidney and 16 h later for prefrontal cortex of brain. *β*-actin was used as an internal control. All values are expressed as mean ± S.E.M. from three independent experiments. Data were analyzed by one-way ANOVA for multiple comparisons and Student-Newman-Keuls test as post hoc test. ^#^ *P* < 0.01 as compared with the vehicle group; *P < 0.05 as compared with the LPS injected group.

The *R*. *rosea* extracts exerted an anti-inflammatory effect on LPS-induced responses accompanied by the expression of proinflammatory cytokines in mice. Kidney and brain prefrontal cortex of ICR mice were collected after 6 h (kidney) or 16 h (brain) of oral administration of *R*. *rosea* extract (500 mg/kg) and LPS treatment. The mRNA expression levels of the iNOS, IL-1*β*, and TNF-*α* were reduced by treatment with *R*. *rosea* extracts (Figure 5). These results indicated that *R*. *rosea* extracts have an anti-inflammatory effect on the expression of LPS-induced proinflammatory cytokines in mice centrally and peripherally.

### 3.4. *Rhodiola rosea* Constituents Suppressed the L-Glu-Induced Neurotoxicity in Primary Cortical Neurons

**Figure 6:**
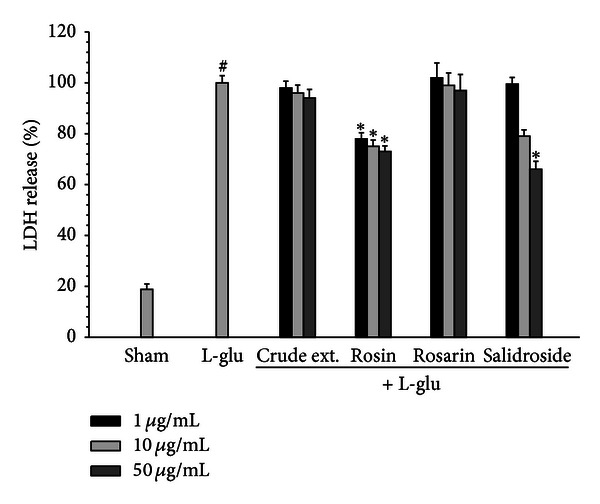
Effects of *R*. *rosea* constituents on L-glutamate toxicity in cultured cortical cells. Neurons were treated by *R*. *rosea* constituents (1, 10, 50 *μ*g/mL) with L-glu (60 *μ*M) for 18 h. Neuronal toxicity was measured by using LDH assay. Data were analyzed by one-way ANOVA for multiple comparisons and Student-Newman-Keuls test as post hoc test. All values are expressed as mean ± S.E.M. from three independent experiments. ^#^ *P* < 0.01 as compared with the vehicle group; *P < 0.05 as compared with the L-glu-treated group.

To investigate the antineurotoxic effect of constituents of *R*. *rosea*, the L-glutamate-induced neurotoxicity was measured after treating cortical neuronal cells with L-glutamate (60 *μ*M) in the presence of *R*. *rosea* constituents for 18 h. The neuronal toxicity was measured by using LDH assay. The neuroprotective effects of constituents of *R*. *rosea*, rosin and salidroside, were evidenced by the significant decrease of LDH release (Figure 6). 

### 3.5. *Rhodiola rosea* Constituents Reduced the L-Glu-Induced Expression of MAPK in Primary Cortical Neurons

**Figure 7:**
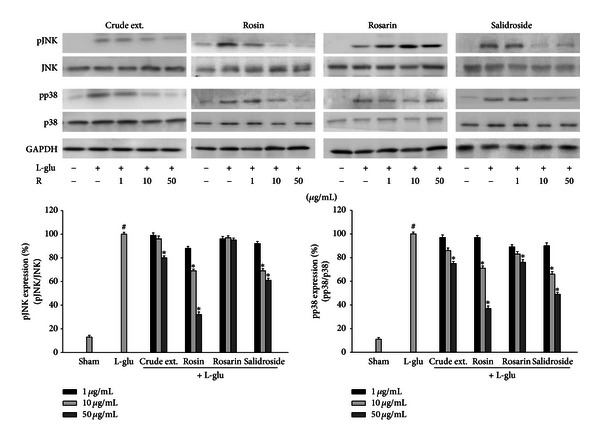
Effects of *R*. *rosea* constituents on the L-glu-induced expressions of MAPK expression in primary neuronal cells. Primary cortical neuronal cell extracts were collected after activation by L-glu (60 *μ*M) with or without treatment of *R*. *rosea* constituents for 18 h and immunoblot analysis was performed using phospho- or total JNK, p38 antibodies. GAPDH was used as an internal control. All values are expressed as mean ± S.E.M. from three independent experiments. Data were analyzed by one-way ANOVA for multiple comparison and Student-Newman-Keuls test as post hoc test. ^#^ *P* < 0.01 as compared with the vehicle group; *P < 0.05 as compared with the L-glu treated group.

*R*. *rosea* exerted an antineurotoxicity effect by the suppression of MAPK. Primary cortical neuronal cells were treated with constituents of *R*. *rosea* and L-glu for 18 h. The elevated levels of the pJNK and pp38 were reduced by crude extract and constituents of *R*. *rosea*, rosin and salidroside, in a dose-dependent manner (Figure 7). These results indicated that rosin and salidroside had an antineurotoxic effect on the suppression of L-glu-induced MAPK expression in primary cortical neurons.

## 4. Discussion

Over the past few decades, many researchers have attempted to develop antineurotoxic agents that are capable of preventing the release of glutamate [27], activation of microglia [28], oxidative stress [29], and apoptosis [30]. Therefore, we also searched for active natural products with better neuroprotective effects and fewer side effects. The main purpose of this study was to determine the anti-inflammatory and neuroprotective effects of *R*. *rosea* constituents in microglial and neuronal cells.

The functional characteristics of microglia have received increasing attention, as these cells play a key role in the inflammatory reaction [31, 32]. Activation of microglia occurred during the development of neurodegenerative pathologies such as Alzheimer's and Parkinson's diseases [33]. In vivo studies also proved that activated microglia produce large amounts of reactive oxygen species (ROS), nitric oxide (NO), and proinflammatory cytokines such as TNF-*α*, interleukin-1*β* (IL-1*β*), and interleukin-6 (IL-6), which, in turn, cause neuronal damage [34–36]. Therefore, treatment with anti-inflammatory drugs can appear to be the most promising option for neurodegenerative disease like Alzheimer's disease. Since nitric oxide is one of the main inflammatory mediators and plays an important role in neuroinflammatory disease, the effect of the *R*. *rosea* constituents on the NO production was investigated in LPS-stimulated microglial cells. The present study also suggested that *R*. *rosea* constituents strongly inhibit NO production and the expression of iNOS, the key enzyme for NO in LPS-stimulated BV2 microglial cells. LPS is a stimulator which is responding to the inflammation to generate TNF-*α*. Currently, the treatment of inflammation-mediated diseases has been studied to suppress the TNF-*α* production which is released by LPS-stimulated microglia [37]. In the following studies, the inhibitory effect of constituents of *R*. *rosea* against TNF-*α*, IL-1*β*, and IL-6 was investigated because these cytokines are also known as major proinflammatory mediators in the progress of neuroinflammatory disease. In these studies, constituents of *R*. *rosea* treatment decreased the production of TNF-*α*, Il-1*β*, and IL-6 which are induced by LPS in BV2 microglia cells in a dose-dependent manner. In particular, TNF-*α*, which may potentiate damage to neuronal cell, is a proinflammatory cytokine and costimulator which is thought to be mediated in the regulation of iNOS gene, predominantly through the mitogen-activated protein kinase (MAPK) and NF-*κ*B signaling pathway [38, 39]. And then regarding the anti-inflammatory role of R. roesa, we determined whether *R*. *rosea* decreased the proinflammatory cytokine expression in mice. This idea is showing that disruption or attenuation of peripheral organ function is linked to an increased risk for chronic inflammatory disease [40–43]. In the present study, oral administration of *R*. *rosea* extract significantly decreases iNOS and proinflammatory cytokines responses in not only kidney but also prefrontal cortex of brain. These results suggest that *R*. *rosea* constituents can be delivered to the brain and suppress the inflammation in the CNS. Further experiment of the anti-inflammatory effect of *R*. *rosea* to determine the signal cascade from TLR-4 which induces a signaling cascade leading to the activation of NF-*κ*B is under investigation.

Alzheimer's disease is a progressive neurodegenerative disease characterized by the presence of two types of abnormal deposits, senile plaques and neurofibrillary tangles, and by extensive neuronal loss [44]. Other studies have suggested the involvement of glutamate cytotoxicity in various neurodegenerative diseases [45] and that amyloid-*β* increases the vulnerability of cultured cortical neurons to glutamate cytotoxicity [20]. Thus, glutamate may play an important role in amyloid *β*-induced cytotoxicity in the cerebral cortex. Therefore, we investigated the modulation of signaling pathways in the neurotoxic conditions induced by L-glutamate treatment with *R*. *rosea*. L-glutamate release and subsequent excitotoxic cell damage has been proposed as a major mechanism producing neuronal cell death in several experimental paradigms of human neurodegenerative disorders [46]. Examination of the intracellular mechanism of neuroprotection against acute L-glu-induced neurotoxicity demonstrated that *R*. *rosea* treatment protects against L-glu-induced neurotoxicity. 

In AD brain, the association between neurotoxicity and MAPK activation has been observed in dystrophic neuritis and astroglial cells [47]. Neurotoxicity is one of the major stimuli for MAPK cascades which are involved in the apoptotic signal transduction. It has been reported that the activation of JNK and p38 MAPK is closely related with cytotoxicity by insults, while the activation of ERK is associated with cell proliferation and serves as an anti-apoptotic signal [48]. In fact, regulators upstream of ERK are distinct from those involved in JNK and p38 activation. 

In the present study, *R*. *rosea* constituents inhibit L-glu-induced JNK and p38 MAPK, but not ERK phosphorylation (data not shown). Further study on whether L-glu could stimulate a rapid, transient activation of ERK in cortical neuronal cells, and whether *R*. *rosea* has any effects on expression is now under investigation. 

In summary, *R*. *rosea* constituents could ameliorate the inflammation and neurotoxicity in cortical neuronal cells. The protective effects of *R*. *rosea* constituents not only were related to modulate endogenous anti-inflammatory, but also affected the neuronal over activation. As far as we know, this is the first report to demonstrate that *R*. *rosea* has the neuroprotective effects against L-glu-induced neurotoxicity in cortical neuronal cells. The protective effects of *R*. *rosea* against neurotoxicity may provide the pharmacological basis of its clinical usage in the treatment of neurodegenerative diseases.
